# Poisoning by Sulphuric Acid

**Published:** 1847-04

**Authors:** 


*Poisoning by Sulphuric Acid.*—On Monday, Dec. 29th, 1845, a boy, aged 9, was admitted into the London Hospital, under Dr. Little, suffering from the effects of oil of vitriol. He was at play in the street, when a strange boy give him a teaspoonful of it to drink; he swallowed about an ounce of the acid, and was instantly seized with excruciating pains in his throat and stomach. He ran home, and, according to his father's account, looked as if somebody had been smearing his lips with white paint. He was carried as quickly as possible to a neighbouring surgeon, who administered a great quantity of chalk and magnesia; five minutes, however, must have elapsed before anything was done. His father then brought him to the hospital, and he became very sick, throwing up a quantity of brownish matter, from which the chalk readily subsided, leaving a supernatant liquor, strongly resembling porter; it was chiefly mucous, charred by the sulphuric acid. He appeared to be suffering a great deal, the countenance was anxious and deadly pale, the extremities cold, and the pulse scarcely perceptible. Under active treatment he rallied, and in about seven hours fell into a quiet sleep; he passed a very comfortable night, and got out of bed twice to void his urine. In the morning, the countenance still looked anxious; he complained of pain in his throat and stomach, though it was not increased by pressure; the lips were swollen, and surrounded by reddish-brown scabs, the effects of the acid; the tongue also was covered with a pearly-white epithelium, which had already begun to peel off from its sides, the skin was hot and dry; the pulse one hundred and thirty-two, hard and jerking. He was ordered albuminous food, and two leeches were applied over the top of the sternum. This febrile condition lasted for four days, the skin remaining hot and dry, and the pulse quick and jerking; during this time he was copiously salivated, and he spat up a great deal of white, shreddy epithelium; the urine was high coloured and increased in quantity, while the bowels remained obstinately constipated. On the fifth day an active purgative relieved them, and from that time he gradually got well. The urine which he voided during the first four days of his illness was submitted to chemical analysis. Its quantity was greater than natural; it had a deep red colour, a strong acid reaction, and a specific gravity varying from 1046 to 1030; it did not contain the least trace of albumen, but held so much of the triple phosphates in solution; that on neutralizing with ammonia they were thrown down in the form of a copious white precipitate. On testing for sulphuric acid, there was found, on the first day, a quantity equal to sixty-two grains of the strongest oil of vitriol; on the second day, forty grains; on the third day, 18.3 grains, and on the fourth the equivalent of 12.7 grains. The urine was not examined on the following day, but on the sixth it had again become natural. In all, therefore, 133 grains of monohydrated sulphuric acid were thus got rid of—a quantity which is about equal to an ounce and three-quarters of the dilute acid of the London Pharmacopœia.

The case offers the following peculiarities:

1. That he should have recovered after the poison had remained so long in the stomach.
2. Its detection in the urine shows that the acid had been absorbed.
3. Its action was almost entirely local, the only constitutional symptoms being a little fever with copious diuresis.

4. That the duty of getting rid of the poison rested with the kidneys.

5. That as much as, or rather the equivalent of, 133 grains of the strongest oil of vitriol should be permitted to circulate with the blood of a child nine years old without producing any very dangerous consequences.—*Lond. Med. Gaz.*

